# Chloroplast genome sequence of triploid *Toxicodendron vernicifluum* and comparative analyses with other lacquer chloroplast genomes

**DOI:** 10.1186/s12864-023-09154-2

**Published:** 2023-01-31

**Authors:** Dan Zong, Zhensheng Qiao, Jintao Zhou, Peiling Li, Peihua Gan, Meirong Ren, Chengzhong He

**Affiliations:** 1 Key Laboratory for Forestry Resources Conservation and Utilization in the Southwest Mountains of China, Ministry of Education, Southwest Forestry University, Kunming, China; 2 Key Laboratory for Forest Genetics and Tree Improvement & Propagation in Universities of Yunnan Province, Southwest Forestry University, Kunming, China; 3 Key Laboratory of Biodiversity Conservation in Southwest China, State Forestry Administration, Southwest Forestry University, Kunming, China

**Keywords:** Triploid *T. vernicifluum*, Chloroplast genome, Phylogeny analyses, SSR markers

## Abstract

**Background:**

*Toxicodendron vernicifluum*, belonging to the family Anacardiaceae, is an important commercial arbor species, which can provide us with the raw lacquer, an excellent adhesive and painting material used to make lacquer ware. Compared with diploid, triploid lacquer tree has a higher yield of raw lacquer and stronger resistance to stress*.* Triploid *T. vernicifluum* was a newly discovered natural triploid lacquer tree. However, the taxonomy of triploid *T. vernicifluum* has remained uncertain. Here, we sequenced and analyzed the complete chloroplast (cp) genome of triploid *T. vernicifluum* and compared it with related species of *Toxicodendron* genus based on chloroplast genome and SSR markers.

**Results:**

The plastome of triploid *T. vernicifluum* is 158,221 bp in length, including a pair of inverted repeats (IRs) of 26,462 bp, separated by a large single-copy region of 86,951 bp and a small single-copy region of 18,346 bp. In total, 132 genes including 87 protein-coding genes, 37 tRNA genes and 8 rRNA genes were identified in the triploid *T. vernicifluum*. Among these, 16 genes were duplicated in the IR regions, 14 genes contain one intron, while three genes contain two introns. After nucleotide substitutions, seven small inversions were analyzed in the chloroplast genomes, eight hotspot regions were found, which could be useful molecular genetic markers for future population genetics. Phylogenetic analyses showed that triploid *T. vernicifluum* was a sister to *T. vernicifluum* cv. Dahongpao and *T. vernicifluum* cv. Hongpigaobachi. Moreover, phylogenetic clustering based on the SSR markers showed that all the samples of triploid *T. vernicifluum*, *T. vernicifluum* cv. Dahongpao and *T. vernicifluum* cv. Hongpigaobachi in one group, while the samples of *T. vernicifluum* and *T. succedaneum* in another group, which is consistent with the cp genome and morphological analysis.

**Conclusions:**

The current genomic datasets provide pivotal genetic resources to determine the phylogenetic relationships, variety identification, breeding and resource exploitation, and future genetic diversity-related studies of *T. vernicifluum*.

**Supplementary Information:**

The online version contains supplementary material available at 10.1186/s12864-023-09154-2.

## Background

Polyploidy, that is having multiple sets of chromosomes as a consequence of whole-genome duplication, is common in nature and provides a major mechanism for adaptation and speciation [1, 2]. The polyploidy genotypes may lead to differences in morphology, physiology and molecular characteristics, etc [3]. *Toxicodendron vernicifluum*, belonging to the family Anacardiaceae, is a deciduous tree species with a toxic sap [4]. The resin and sap which was extracted from lacquer trees were used as paint for culture assets and lacquer wares, making it a natural material of cultural and social significance [5–7]. In addition, lacquer tree is also used as a food additive, natural dye, or in herbal medicine to improve blood circulation and to prevent blood stasis, while the metabolic extract of the leaves has neuroprotective and anti-inflammatory activity [8]. The economic value of different varieties of lacquer can be judged by lacquer yield, the growth rate of lacquer and genetic diversity [9].

Compared with the diploid lacquer tree, the triploid lacquer tree has a higher yield of raw lacquer and stronger resistance to stress [10]. *T. vernicifluum* cv. Dahongpao (2n=3x=45) was the first nature triploid lacquer tree which was discovered at Bashan Mountain in Shaanxi Province, China and now is widely introduced into the planting area of lacquer tree [11, 12]. Han et al [13] determined the pseudo-polyploidy of *T. vernicifluum* was a natural triploid lacquer (2n=3x=45) by observation of the stomatal characteristic of leaf lower epidermis, analysis of lower epidermis, flow cytometry and measurement of genome size, and it was named as triploid *T. vernicifluum*. However, as an economic tree species, *T. vernicifluum* has been widely introduced and cultivated [14], which lead to the taxonomy classification was difficult to resolve because of considerable phenotypic variability with overlapping morphologies.

Chloroplast (cp) genomes have assembled notable contributions in diverse plant families, setting evolutionary within phylogenetic clades [15–17], because the lack of recombination and maternal transmission means the chloroplast genome is also useful for tracking source populations [18–20] and chloroplast genomes variation could provide valuable genetic markers for the analysis of polyploids [21]. In addition, the cp genome plays an important role in reconstruction of the plant phylogeny and understanding the origins of economically important cultivated species and changes that have taken place during domestication [22]. Microsatellites are powerful markers to detect genetic variation, because of their high mutation rate and high polymorphism, allowing us to distinguish between closely related individuals, and thus to assess parentage and kinship relationships [23].

In this study, we reported the characteristics of the complete chloroplast genome sequences of triploid *T. vernicifluum* for the first time and compared with other *Toxicodendron* cp genomes to investigate the relationship among chloroplast genome. To understand the relationships of the triploid *T. vernicifluum*, we constructed the phylogenetic tree using their fully sequenced chloroplast genome sequenced and SSR molecular markers. The results will provide a theoretical basis for variety identification, breeding and resource exploitation.

## Results

### Morphological traits of five *Toxicodendron* accessions

The leaf length, leaflet number, leaflet length and leaflet width were compared among the five accessions (Table 1), the results showed that the leaf length of triploid *T. vernicifluum* (TZT) was significantly higher than other four accessions. The leaflet number, leaflet length and leaflet width were significantly lower than *T. vernicifluum* cv. Dahongpao (DHP) and *T. vernicifluum* cv. Hongpigaobachi (GBC), and higher than *T. vernicifluum* (TZG) and *T. succedaneum* (TRB). The leaf shape index of TZT, DHP and GBC (2.03, 2.11 and 2.18, respectively) were lower than TZG (3.21) and TRB (3.31). In addition, all the morphological traits both showed that there were no significant difference between *T. vernicifluum* cv. Dahongpao (DHP) and *T. vernicifluum* cv. Hongpigaobachi (GBC).

**Table 1:** Leaf morphological characteristics of five *Toxicodendron* accessions

**Accessions**	**Leaf length**	**Leaflet number**	**Leaflet length**	**Leaflet width**	**Leaf shape index**
TZT	33.67±4.19^A^	8.56±0.92^C^	11.29±1.52^B^	5.60±0.62^B^	2.03±0.17^B^
DHP	29.41±5.97^B^	10.97±1.73^AB^	12.97±1.83^A^	6.21±1.00^A^	2.11±0.20^B^
GBC	31.02±6.93^AB^	10.04±1.78^B^	14.38±2.31^A^	6.62±0.54^A^	2.18±0.35^B^
TZG	25.17±4.17^C^	10.93±2.52^AB^	9.77±1.81^D^	3.05±0.42^C^	3.21±0.37^A^
TRB	21.08±3.70^D^	12.22±2.76^A^	9.28±1.42^D^	2.86±0.56^C^	3.31±0.48^A^

Values are the means ± SD of triplicate assays. Data in columns with the different capital letters are significantly different *P*<0.01

*TZT* triploid *T. vernicifluum*, *DHP T. vernicifluum*cv. Dahongpao, *GBC*: *T. vernicifluum* cv. Hongpigaobachi, *TZG T. vernicifluum*, *TRB T. succedaneum*

### General features of *Toxicodendron* chloroplast genome

**Fig. 1:**
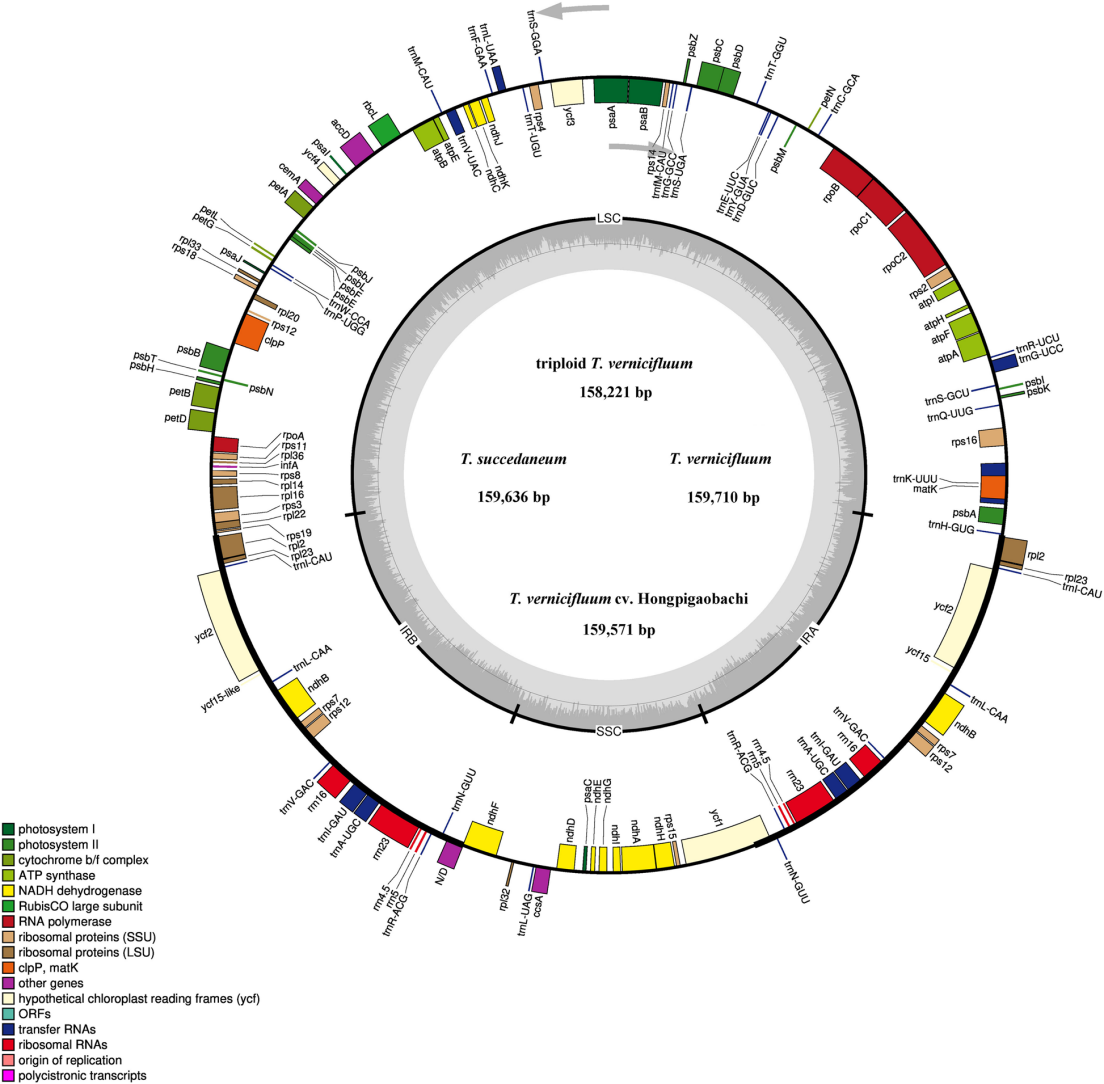
Gene map of the four new sequenced *Toxicodendron* cp genomes. Genes shown outside the outer circle are transcribed clockwise and those inside are transcribed counterclockwise. Genes belonging to different functional groups are color-coded. The dashed area in the inner circle indicates the GC content of the cp genome of *Toxicodendron*

The results showed that the cp genomes of triploid *T. vernicifluum* (TZT), *T. vernicifluum* cv. Hongpigaobachi (GBC), *T. succedaneum* (TRB) and *T. vernicifluum* (TZG) have a typical circular tetramerous structure like other related plants [24, 25], with a genome size of 158,221 bp, 159,571 bp, 159,636 bp and 159,710 bp, respectively. Both the cp genomes comprised of four distinctive parts in which the LSC (86,951 bp, 87,475 bp, 87,523 bp and 87,636 bp) and SSC (18, 346 bp, 19,074 bp, 19,045 bp and 19, 041 bp) are separated by two IRs (26,462 bp, 26,511 bp, 26,534 bp and 26,525 bp) (Fig. 1, Table 2).

**Table 2:** Features of ten *Toxicodendron* cp genomes

Genome Feature	TZT	GBC	TRB	TZG	DHP	Ts	Tg	Tsy	Tdi	Tyg
Length/bp	158,221	159,571	159,636	159,710	159,571	159,710	159,613	159,600	159,543	159, 571
LSC/bp	86,951	87,475	87,523	87,636	87,475	87,622	87,227	87,630	87,693	87,475
IR/bp	26,462	26,511	26,534	26,525	26,511	26,524	26,490	26,525	26,525	26,511
SSC/bp	18,346	19,074	19,045	19,041	19,074	19,040	18,911	19,040	18,797	19,074
GC/%	38.0	38.0	37.9	37.9	38.0	37.9	37.9	37.9	38.0	37.9
GC in LSC/%	36.1	36.1	36.0	36.0	36.1	36.0	36.0	36.0	36.1	36.0
GC in IR/%	42.9	43.0	43.0	42.9	43.0	42.9	43.0	42.9	42.9	43.0
GC in SSC/%	32.7	32.6	32.4	32.5	32.6	32.5	32.6	32.5	32.7	32.6
Number of Genes	132	132	132	132	132	132	132	132	132	132
Number of protein coding genes	87	87	87	87	87	87	87	87	87	87
Number of rRNA	8	8	8	8	8	8	8	8	8	8
Number of tRNA	37	37	37	37	37	37	37	37	37	37
Genes with intron	22	22	22	22	22	22	22	22	22	19
GenBank Number	OP271782	OP279729	OP235457	OP279730	MK550621	MT211614	MT269874	MT211615	OP585546	MK419151

*TZT* triploid *T. vernicifluum*, *DHP T. vernicifluum* cv. Dahongpao, *GBC T. vernicifluum* cv. Hongpigaobachi, *TRB T. succedaneum*, *TZG T. vernicifluum Ts T. succedaneum*, *Tg T. griffithii*, *Tsy T. sylvestre*, *Tdi T. diversilobum*, *Tyg T. vernicifluum* cv. Yanggangdamu

The four cp genomes were analyzed and compared with *T. succedaneum* (Ts), *T. griffithii* (Tg), *T. sylvestre* (Tsy), *T. diversilobum* (Tdi), and *T. vernicifluum* cv. Yanggangdamu (Tyg) which belong to the same genus. The nine *Toxicodendron* cp genomes have minor differences in length, except TZT. The size of these cp genomes ranges from 158,221 bp (TZT) to 159,710 bp (TZG and Ts). The size of the IR region ranges from 26,462 bp (TZT) to 26,534 bp (TRB), while the SSC and LSC size varies from 18,346 bp (TZT) to 19,074 bp (DHP, GBC and Tyg) and from 86,951 bp (TZT) to 87,693 bp (Tdi) (Table 2). The overall GC content in the whole genome sequences was practically identical among these plastomes (37.9-38.0%). Furthermore, the GC contents are unevenly distributed across regions of the cp genome, which were found 36.0-36.1%, 42.9-43.0% and 32.4-32.7% for the LSC, IR and SSC regions, respectively (Table 2).

The ten *Toxicodendron* cp genomes encoded the same 132 functional genes, consisting of 87 protein-coding genes, 8 rRNA genes and 37 tRNA genes and a total of 16 genes were duplicated in the IR regions (Table 2 and Table S1). For both accessions, 14 genes (*atpF, ndhA, ndhB, petB, petD, rpl2, rpl16, rpoC1, rps16, trnA-UGC, trnG-UCC, trnI-GAU, trnL-UAA* and *trnV-GAC*) contain one intron, while three genes (*clpP, rps12* and *ycf3*) contain two introns (Table S1)

### SSR polymorphisms and long repeat analyses

The number of cp genome SSRs (cpSSRs) ranged from 52 to 70 among the ten *Toxicodendron* plastomes (Table 3). The number of cpSSRs in the four accessions TZT, DHP, GBC and Tyg were similar (52), and the numbers of cpSSRs in TZG (70) was the same as that in Tsy (70). The mononucleotide repeat (P1) number with the highest variability ranged from 45 (TZT) to 60 (TZG and Tsy), and most of the P1s were composed of poly A and T repeats. Dinucleotide (P2) repeat sequences in the ten accessions were AT or TA repeats. Only one pentanucleotide (P5) repeat was detected in Tdi. Within the ten plastomes, SSR loci were primarily in the LSC region (Table 3 and Table S2). In addition, 6 (TRB, TZG, Ts and Tsy) and 7 (TZT, DHP, GBC, Tg, Tdi and Tsy) SSR loci were detected in the protein-coding genes *rpoC2*, *atpB*, *ycf1*, *ndhI* (only in DHP, GBC and Tyg) and *ndhF* (only in Tg), other situated in intergenic spacers and introns (Table S2)

**Table 3:** Simple sequence repeats (SSRs) in the ten *Toxicodendron* cp genomes

**Accessions**	**SSR loci no.**	**P1**				**P2**		**P5**	**Compound**	**Location**			**Region**		
		**A**	**T**	**C**	**G**	**AT**	**TA**	**TTAAT**		**CDS**	**IGS**	**Intron**	**LSC**	**IR**	**SSC**
TZT	52	15	26	1	3	2	1	/	4	7	39	6	38	8	6
DHP	52	20	25	1	3	1	/	/	2	7	39	4	36	10	6
GBC	52	20	25	1	3	1	/	/	2	7	39	4	36	10	6
TRB	63	20	30	2	2	1	1	/	7	6	49	8	47	10	6
TZG	70	26	30	2	2	1	1	/	8	6	54	10	53	10	7
Ts	69	24	29	2	2	1	1	/	10	6	54	9	52	10	7
Tg	65	24	28	1	1	1	/	/	10	7	51	7	47	8	10
Tsy	70	26	30	2	2	1	1	/	8	6	54	10	53	10	7
Tdi	59	24	25	1	1	1	/	1	6	7	47	5	42	8	9
Tyg	52	20	25	1	3	1	/	/	2	7	41	4	36	10	6

*P1* mononucleotide (momo-), *P2* dinucleotide (di-), *P5* pentanucleotide (penta-), *CDS* protein-coding genes, *IGS* intergenic spacer region

**Fig. 2:**
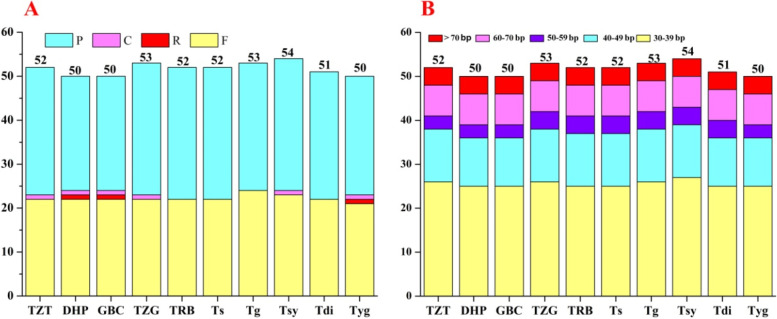
Comparison of long repeats among ten *Toxicodendron* cp genomes. **A** Number of repeats, P: palindromic repeats, R: reverse repeats, C: complement repeats, F: forward repeats. **B** Frequency of each type by length

In the ten *Toxicodendron* plastomes, four repeat types were detected using REputer software (Fig. 2A). We found 50 repeats in DHP (22 F, 1R, 1 C and 26 P), GBC (22 F, 1R, 1 C and 26 P) and Tyg (21 F, 1R, 1 C and 27 P), which was lower than those found in the other seven accessions. 52 repeat (22 F, 1 C and 29 P) was found in the plastomes of TZT. In addition, the majority of repeats varied from 30 to 39 bp in length (Fig. 2B).

### IR expansion and contraction

**Fig. 3:**
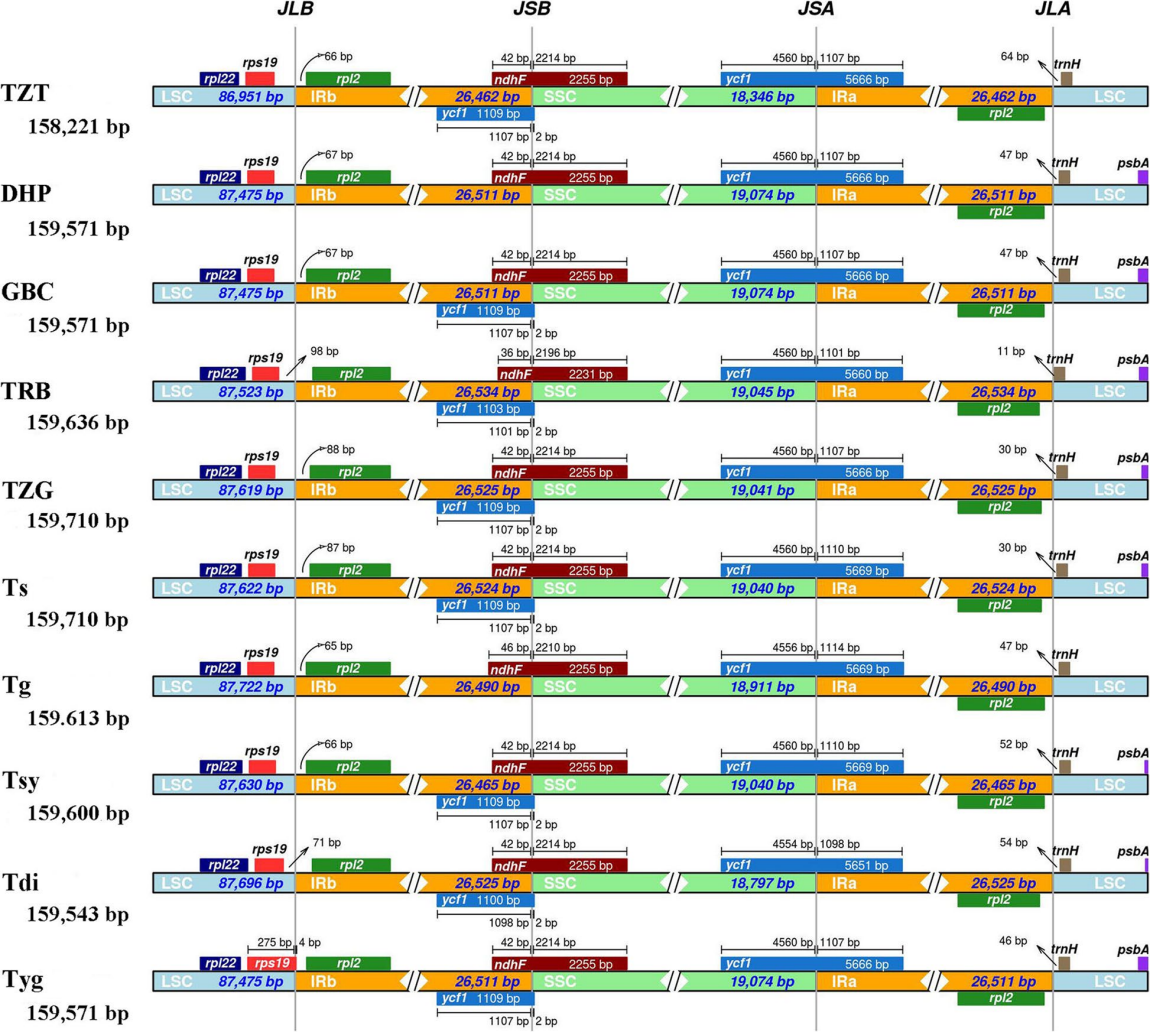
Comparison of the LSC, IR, SSC junction positions among ten *Toxicodendron* cp genomes. JLB: junction of LSC and IRb; JSB: junction of SSC and IRb; JSA: junction of SSC and IRa; JLA: junction of LSC and IRa. Boxes above and below the mainline indicate the adjacent border genes. The gaps between the genes and the boundaries are indicated by the base lengths (bp)

Although cp genomes are highly conserved in terms of genomic structure and size, the IR/SC junction position change caused by expansion and contraction of the IR/SC boundary regions was usually considered as a primary mechanism in creating the length variation of the higher plant cp genomes [26, 27]. The border regions of the ten *Toxicodendron* cp genomes were compared. The IR regions’ lengths correlate across all the compared cp genomes with only slight expansion and contraction (Fig. 3), ranged from 26,462 bp (TZT) to 26,534 bp (TRB) in size, of which *rps19*, *rpl2*, *ycf1*, *ndhF* and *trnH* genes were present at the junctions of the LSC/IR and SSC/IR borders. At the LSC/IRb junction (JLB), the gene *rps19* in the LSC of TZT, DHP, GBC, TRB, TZG, Ts, Tg, Tsy and Tdi contracted 71 to125 bp, whereas Tyg extended 4 bp into the IR region. The gene *rpl2* in the IRa region also contracted by a different number of bases (65-103 bp). The distance between *trnH* and JLA is 11-64 bp. In contrast, the SSC/IR boundary regions were relatively stable. The *ycf1* gene is located at the IRa/SSC (JSA) border in the ten cp genomes, and the junctions of IRa/SSC (JSA) located in *ycf1* within the SSC and IRa regions almost had the same length. The gene *ycf1* in the IRb region and gene *ndhF* in the SSC region interlaced at the IRb/SSC (JSB) border and *ycf1* in the SSC region was astride the border of SSC/IRa (JSA).

### Comparative analysis of genome structure

**Fig. 4:**
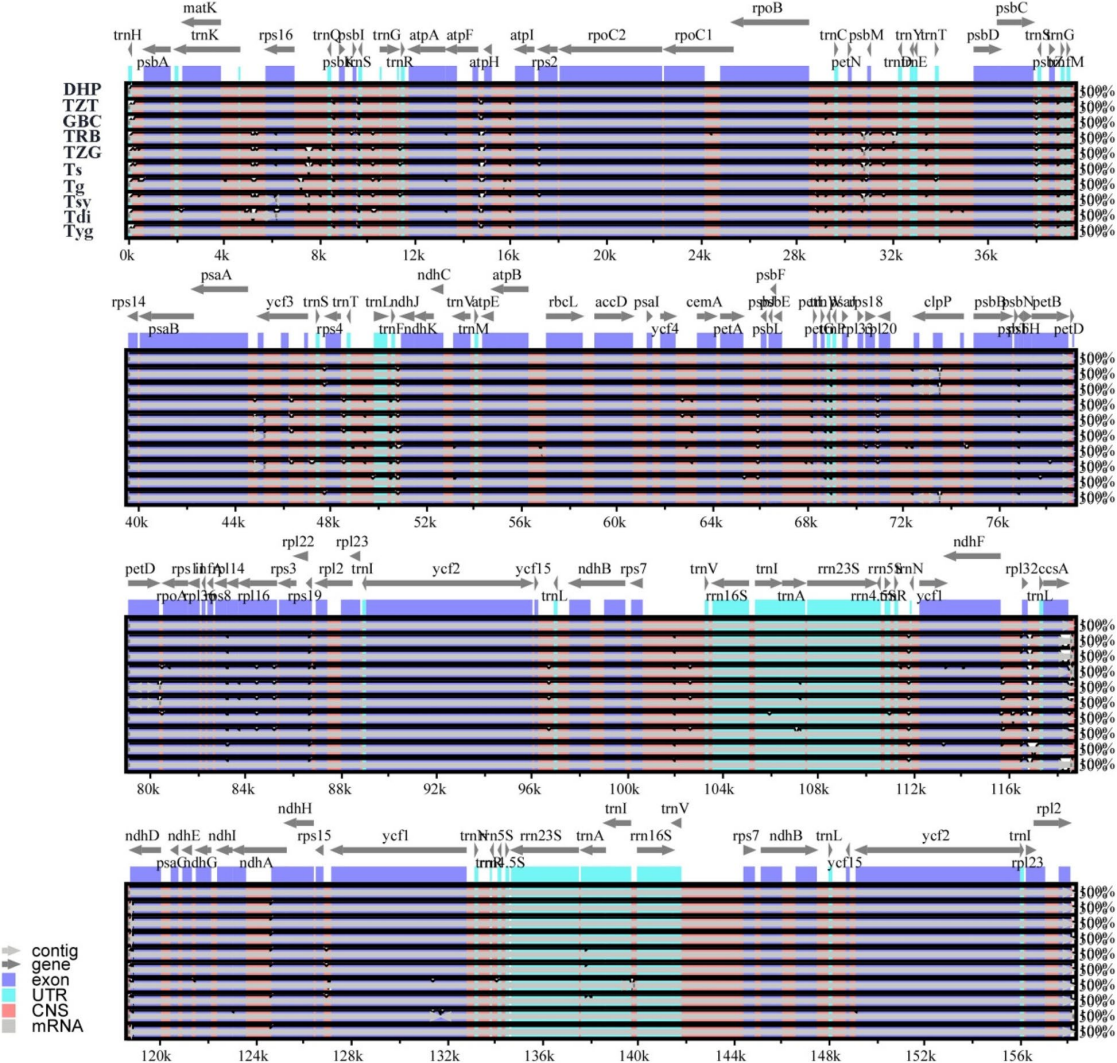
Identity plots comparing the cp genomes of ten *Toxicodendron* accessions using *T. vernicifluum* cv. Dahongpao as a reference sequence. The vertical scale indicates the percentage of identity, ranging from 50 to 100%. The horizontal axis indicates the coordinates within the cp genome. Genome regions are color coded as protein-coding, rRNA, tRNA, intron, and conserved non-coding sequences (CNS)

To investigate the intergeneric divergence of cp genome sequences, the percentage of identity was plotted for ten *Toxicodendron* accessions using mVISTA program with DHP as a reference. The alignment revealed high sequence similarity across the ten cp genomes and no rearrangement occurred (Fig. 4), which suggests that they are highly conserved.

**Fig. 5:**
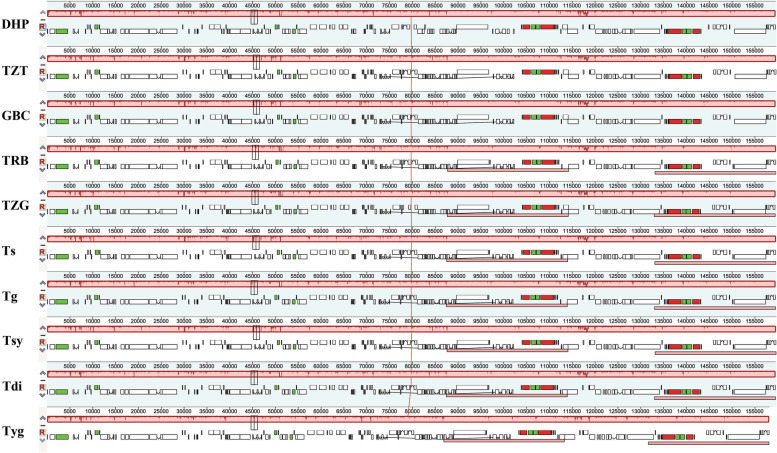
MAUVE alignment of ten *Toxicodendron* accessions cp genomes. The *T. vernicifluum* cv. Dahongpao genome is shown at the top as the reference genome. Within each of the alignments, local collinear blocks are represented by blocks of the same color connected by lines

The cp genome sequences of the ten *Toxicodendron* accessions were aligned by MAUVE, and DHP was used as a reference to compare the gene orders among these cp genomes (Fig. 5). The results showed that all sequences show perfect synteny conservation with no inversion or rearrangements.

**Fig. 6:**
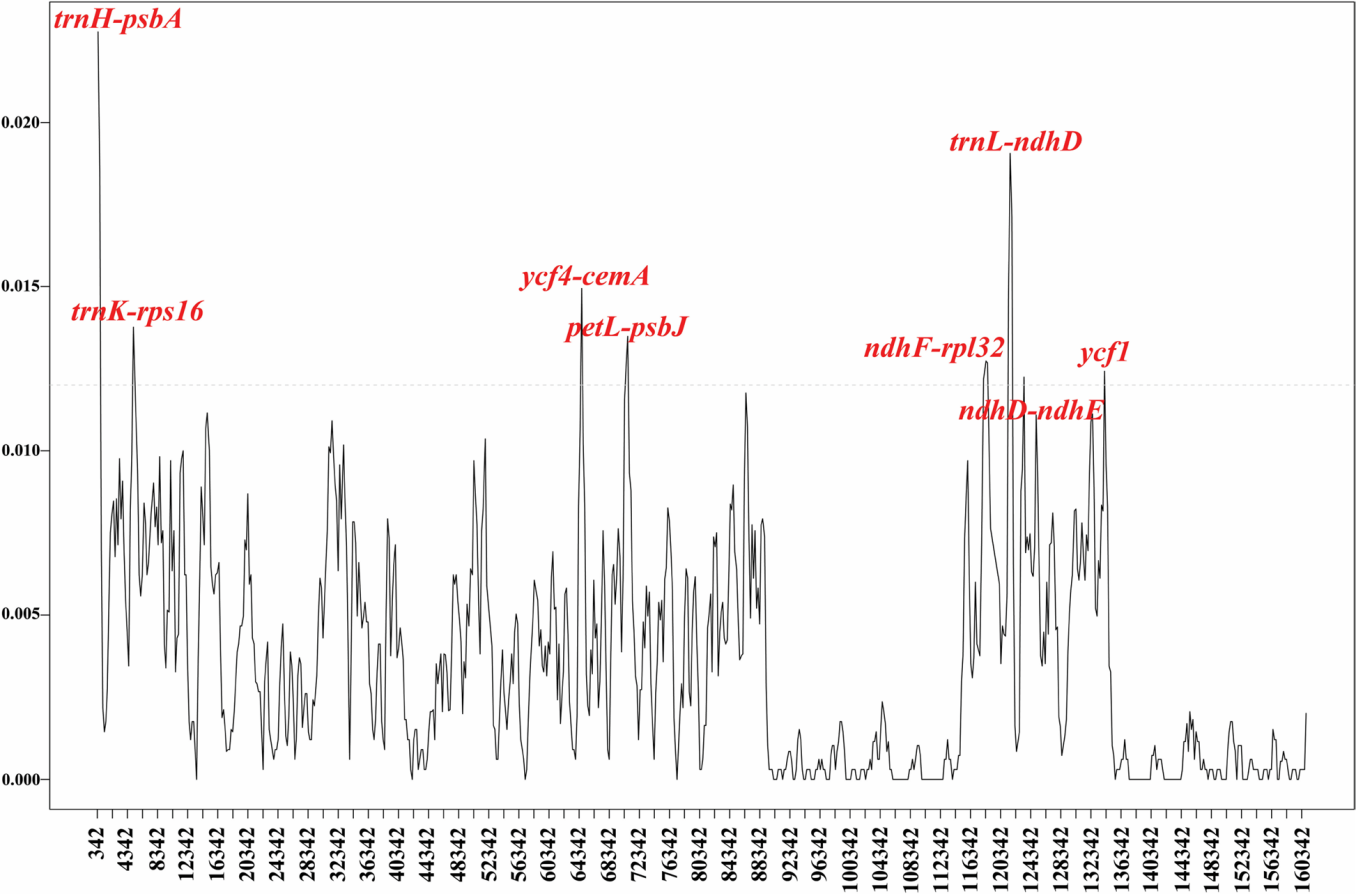
Sliding window analysis of the whole cp genomes of ten *Toxicodendron* accessions. Window length: 600 bp, step size: 200 bp. X-axis, the position of the midpoint of a window; Y-axis, nucleotide diversity of each window

The nucleotide variability (Pi) values of the ten cp genomes were calculated with the DnaSP software. A total of 1794 polymorphic sites were detected and the Pi values ranged from 0.0000 to 0.0240, with the mean of 0.0037. Eight hotspots (Pi>0.0120) were uncovered among the ten cp genomes. They are four intergenic spacers (*trnH-psbA, trnK-rps16*, *ycf4-cemA*, *petL-psbJ*) from the LSC region, three intergenic spacers (*ndhF-rpl32*, *trnL-ndhD* and *ndhD-ndhE*) and one gene regions (*ycf1*) from the SSC regions (Fig. 6). The results indicated that the non-coding regions exhibited more variations than the coding regions. The sequences of these highly variable regions could be developed as barcodes for species identification, phylogenetic analysis, and population genetics research in *Toxicodendron*.

We investigated SNPs, the most abundant type of mutation, in the ten cp genomes, with *T. vernicifluum* cv. Dahongpao (DHP) as the reference. In the gene-coding regions, we detected two SNPs in the comparative combination of GBC-DHP, including one transition (Ts) and one transversion (Tv) SNPs, one Ts were detected in Tyg-DHP, as well as 121 (67 Ts and 54 Tv), 227 (114 Ts and 113 Tv), 102 (46 Ts and 56 Tv), 239 (119 Ts and 120 Tv), 209 (111 Ts and 98 Tv), 244 (124 Ts and 120 Tv) and 199 (95 Ts and 104 Tv) SNPs were detected in the combinations of TZT-DHP, TZG*-*DHP, TRB*-*DHP, Ts-DHP, Tg-DHP, Tsy-DHP and Tdi-DHP (Table 4). Furthermore, 5 (4 Ts and 1 Tv), 260 (96 Ts and 164 Tv), 462 (181 Ts and 281 Tv), 126 (10 Ts and 116 Tv), 462 (177 Ts and 285Tv), 430 (145 Ts and 285 Tv), 463 (181 Ts and 282 Tv), 324 (118 Ts and 206Tv) and 1 (1 Tv) SNPs were detected in noncoding regions among the nine comparative combinations, respectively (Table S3).

**Table 4:** Transitions (Ts) and transversions (Tv) in the protein-coding regions of the nine plastomes, compared with DHP

**Treat**	**Ts**		**Tv**				Total
	A-G	C-T	A-T	A-C	T-G	G-C	
GBC-DHP	0	1	0	1	0	0	2
TZT-DHP	31	36	5	22	19	8	121
TZG-DHP	54	60	7	34	63	9	227
TRB-DHP	22	24	6	12	28	10	102
Ts-DHP	60	59	9	40	62	9	239
Tg-DHP	48	63	12	28	46	12	209
Tsy-DHP	65	59	9	35	66	10	244
Tdi-DHP	44	51	7	35	52	10	199
Tyg-DHP	0	1	0	0	0	0	1

**Fig. 7:**
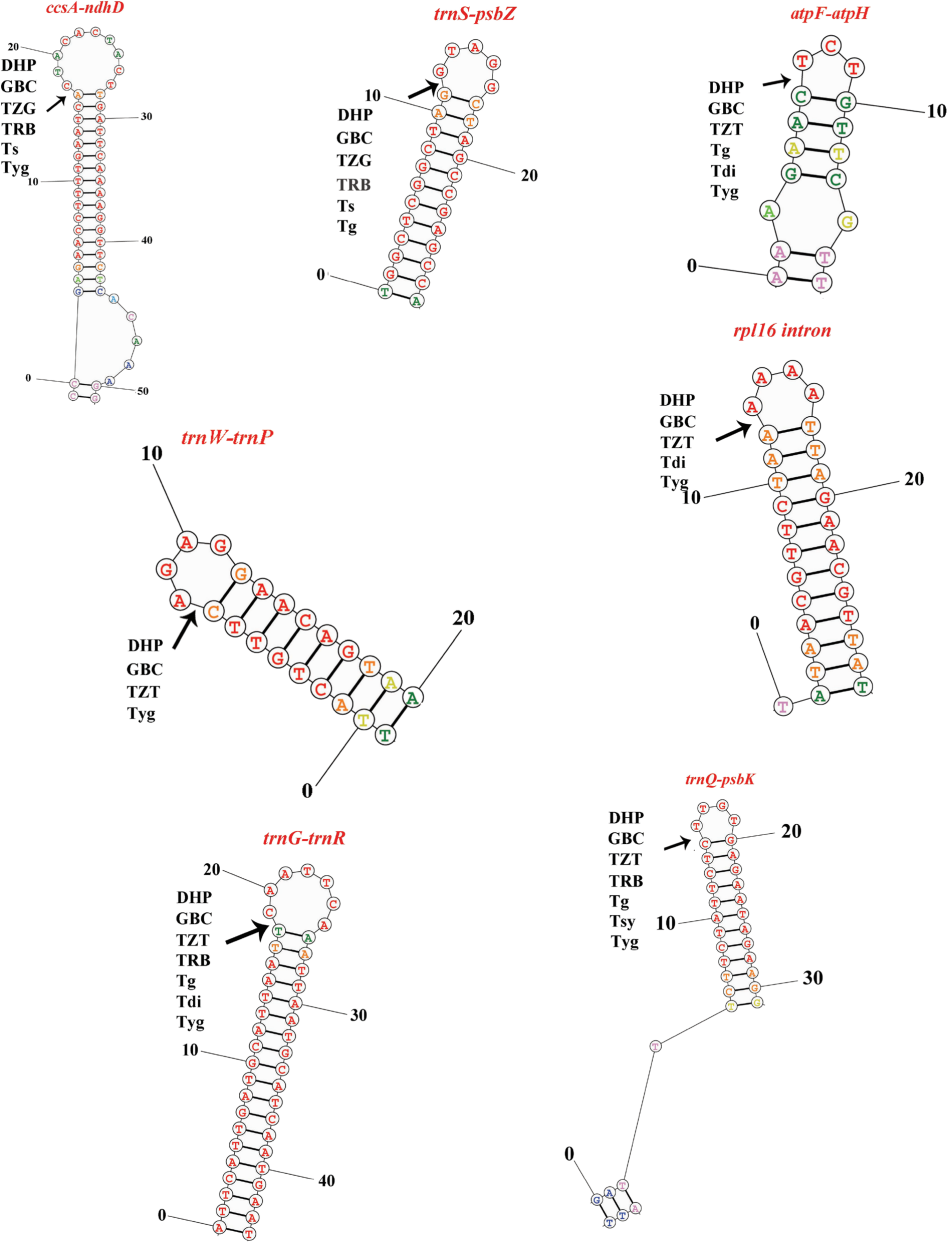
Predicted hairpin loops of small inversions in the ten plastomes of *Toxicodendron*. The arrows in the figure indicate the break points in inversion events

It has been reported that each small inversion is commonly associated with a hairpin secondary structure in the chloroplast genomes [26, 28]. Small inversions are generally detected by performing pairwise comparisons between sequences of closely related taxa [26]. Seven small inversions were identified in the *Toxicodendron* cp genomes and their inverted repeating flanking sequences formed stem-loop structures (Fig. 7). All the inversions were located in noncoding regions including 6 in intergenic spacers (*ccsA-ndhD*, *trnS-psbZ*, *atpF-atpH*, *trnW-trnP*, *trnG-trnR* and *trnQ-psbK*) and one in intron regions (*rpl16* intron). Among the seven small inversions, six were located in LSC region, one in SSC region (*rpl16* intron). The small inversions from *trnW-trnP* occurred only in DHP, GBC, TZT and Tyg. The small inversions from *atpF-atpH*, *trnW-trnP*, *trnG-trnR* and *trnQ-psbK* and *rpl16* intron have been occurred in DHP, GBC and TZT.

### Phylogenetic analysis based on the chloroplast complete genome and SSR molecular markers

**Fig. 8:**
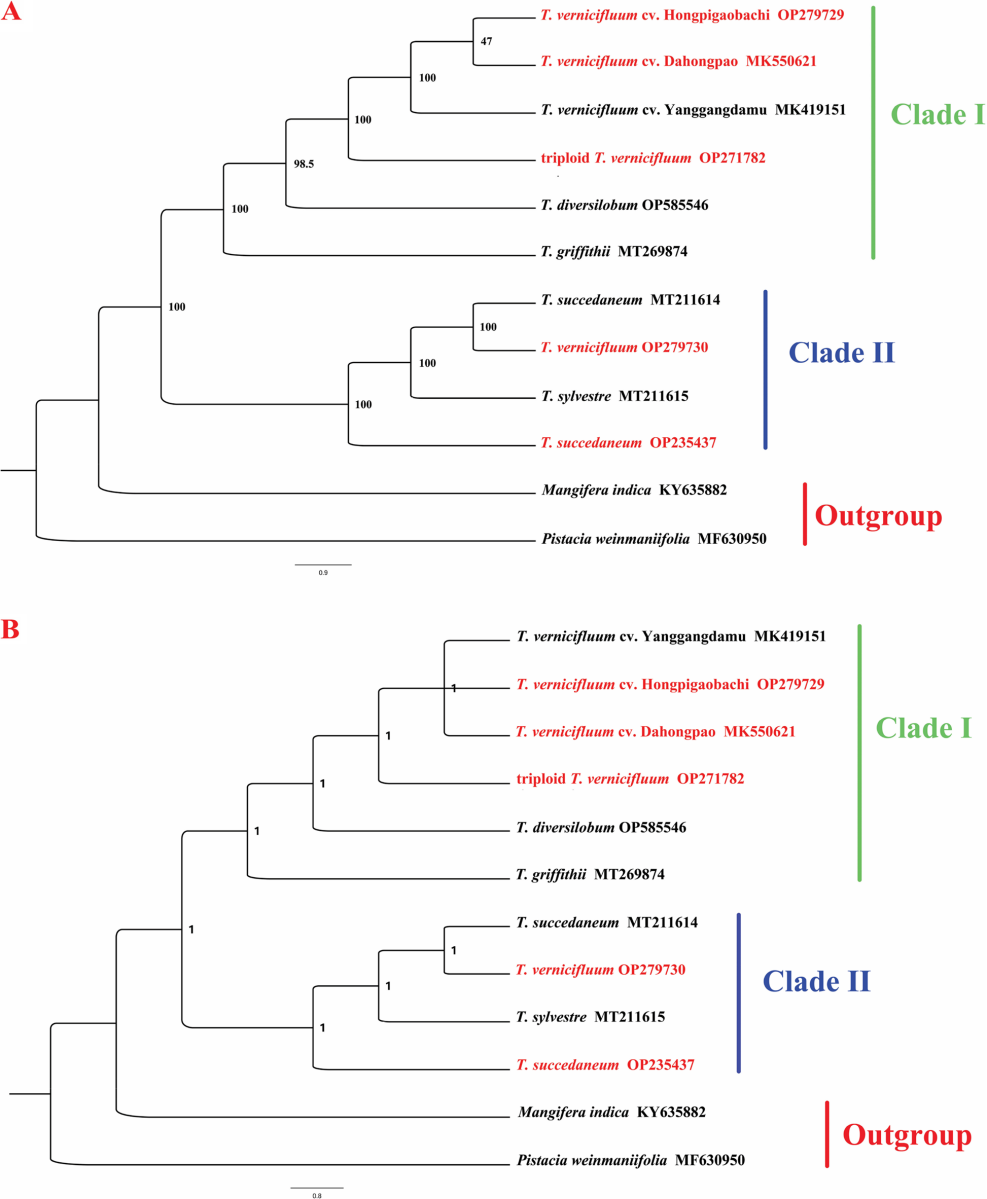
Phylogeny of ten *Toxicodendron* accessions inferred from ML (**A**) and BI (**B**) analyses of different cp genome sequences. Numbers in the clade represent bootstrap (BP) and posterior probability (PP) values

To identified the phylogenetic position of triploid *T. vernicifluum* in *Toxicodendron*, we used 12 cp genomes to phylogenetic analyse. Maximum likelihood (ML) and Bayesian inference (BI) were used to construct phylogenetic tree with *Pistacia weinmaniifolia* and *Mangifera indica* as outgroup (Fig. 8). The topologies of the ML and BI trees were nearly identical, which both showed that *Toxicodendron* species formed a monophyletic clade (BS=100, PP=1). The ten *Toxicodendron* species were divided into two main clades. Clade I contained six accessions (*T. vernicifluum* cv. Dahongpao, *T. vernicifluum* cv. Hongpigaobachi, *T. vernicifluum* cv. Yanggangdamu, triploid *T. vernicifluum*, *T. diversilobum* and *T. griffithii*) and the results showed that *T. vernicifluum* cv. Dahongpao is closely related to *T. vernicifluum* cv. Hongpigaobachi and sister to triploid *T. vernicifluum* and *T. vernicifluum* cv. Yanggangdamu. Clade II consists of *T. succedaneum* (MT211614), *T. vernicifluum*, *T. sylvestre* and *T. succedaneum* (OP235437). The phylogenetic tree was very helpful for us to understand the phylogenetic relationship among more *Toxicodendron* species.

**Fig. 9:**
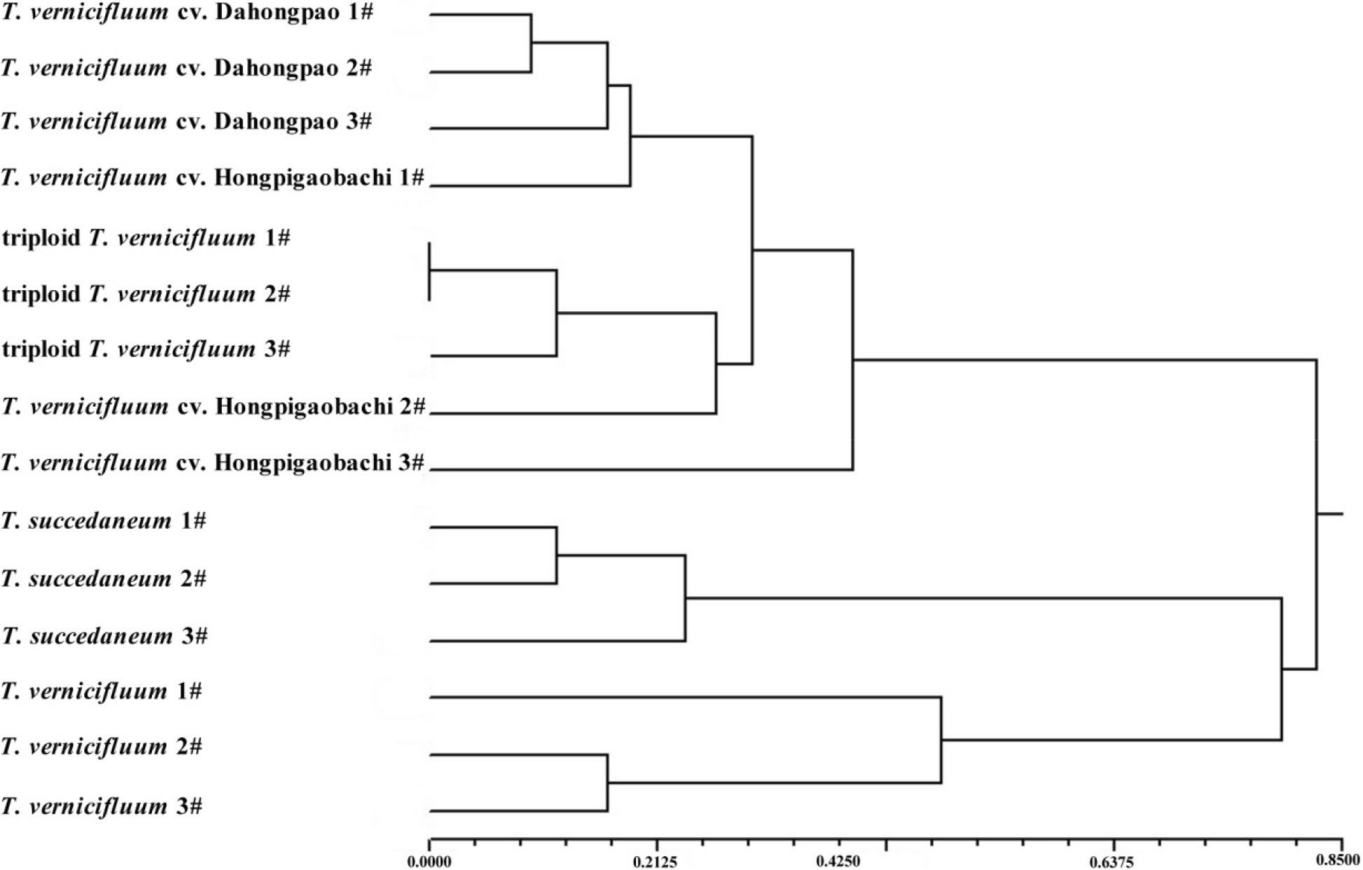
Dendrogram generated by UPGMA from genetic distance data based on SSR markers

Genetic distance among the 15 samples was calculated according to the software GenoDive (Table S4) [29]. Based on Nei’s genetic distance coefficient, a dendrogram was obtained using UPGMA cluster analysis. With this result, two groups could be distinguished. The first group was further divided into two subgroups, three samples of *T. succedaneum* (*T. succedaneum* 1#, *T. succedaneum* 2# and *T. succedaneum* 3#) were included in subgroup 1, and the three samples of *T. vernicifluum* (*T. vernicifluum* 1#, 2# and 3#) were included in the subgroup II. Group II included three accessions of *T. vernicifluum* cv. Dahongpao, *T. vernicifluum* cv. Hongpigaobachi and triploid *T. vernicifluum*, and *T. vernicifluum* cv. Dahongpao was sister to triploid *T. vernicifluum* (Fig. 9).

## Discussion

There are obvious differences in leaf morphology between diploid and polyploidy, which is one of the simpler methods to identify plant ploidy by morphological differences [30]. Leaf shape index (length/width) is an important parameter of leaf shape. The larger the leaf shape index of plant is, the longer and narrower the leaves are, while the smaller the leaf shape index is, the rounder the leaves are [31]. Liu et al [32] used the observational method and paraffin section technique to analyze the intraspecific differences in four kinds of *Toxicodendron* species and the results showed that the sharp differences presented in height, shape, bark and leaves of the four varieties. In this study, morphological traits of five *Toxicodendron* accessions were determined and the leaf length of triploid *T. vernicifluum*, *T. vernicifluum* cv. Dahongpao and *T. vernicifluum* cv. Hongpigaobachi were significantly higher than *T. vernicifluum* and *T. succedaneum*. Therefore, the morphological characteristics of diploid and triploid can be distinguished by the leaf shape characteristics [33]. In addition, all four morphological characteristics (leaf length, leaflet number, leaflet length and width) were shown that *T. vernicifluum* cv. Dahongpao and *T. vernicifluum* cv. Hongpigaobachi had no significant difference, and there were no significant difference between *T. vernicifluum* and *T. succedaneum* in the morphological traits of leaflet number, leaflet length and width. In conclusion, certain accessions are similar in terms of morphological characteristics.

The chloroplast genomes of plants are a valuable resource for developing molecular markers to study intra-species and interspecies evolution [34, 35]. In this study, we sequenced and annotated the cp genomes of four *Toxicodendron* accessions and compared their characteristics with those of six other related species from *Toxicodendron*. Like other angiosperms, *Toxicodendron* cp genome is a circular double-standed DNA molecular, exhibiting a conserved quadripartite structure [24, 25, 36], and the ten cp genomes were relatively conserved, which is consistent with the slow rates of sequence and structure evolution of plant plastomes [37, 38]. The cp genomes ranged from 158,221 bp (triploid *T. vernicifluum*) to 159,710 bp [*T. vernicifluum* and *T. succedaneum* (Ts)], with a difference in size of 1489 bp. Large variation in LSC length (742 bp) accounted for most of this genome-wide variation, which indicated that variation in genome length appears to be mainly caused by variation in LSC length [39]. The GC content of the ten *Toxicodendron* accessions ranged from 37.9-38.0%, which is closely related to species affinity [40]. High GC content is conducive to the stability of the genome and maintaining the complexity of the sequence. The four rRNAs genes have high GC content, which results are a high GC content in the IR regions [41]. Usually, a higher GC content indicated a more stable genome sequence [42].

The expansion and contraction of IR and SC boundaries are thought to be the main cause of CP genome size changes, although CP genomes in plants are highly conserved [37]. The change of the IR and SC junction is a common phenomenon and plays an important role in evolution [43, 44]. After comparing CP genomes among the ten *Toxicodendron* accessions in this study, changes in the IR (26,462-26,534 bp) and boundary between the IR and LSC or SSC of the cp genome of ten accessions were found to be small. Some variations were observed in these cp genomes, mainly due to variation in the LSC regions rather than contraction and expansion of IR region [45, 46]. Comparison between IR regions and the chloroplast genomes of *T. vernicifluum* cv. Dahongpao and *T. vernicifluum* cv. Hongpigaobachi showed similarity, which was the same as for the boundary gene of LSC/IRa, SSC/IRa and LSC/IRb regions and the size of genes and fragments in two adjacent regions. Therefore, we speculate that the genetic relationship between *T. vernicifluum* cv. Dahongpao and *T. vernicifluum* cv. Hongpigaobachi is closer than other accessions, and our subsequent phylogenetic analysis validates our inference.

Previous studies have shown that tRNA activity may be a key factor triggering the inversions events [47]. Small inversions in the cp genome of angiosperms are ubiquitous and commonly associated with a hairpin secondary structure and are generally detected by performing pairwise comparisons between sequences of closely related taxa [26]. Many small inversions are generated by parallel or back mutation events during chloroplast genome evolution [26, 48]. In the present study, seven small inversions were discovered based on the sequence alignment of the ten cp genomes and the inversion in *ccsA-ndhD* have been reported in other studies [16, 49–51]. *trnW-trnP* were specific in *T. vernicifluum* cv. Dahongpao and *T. vernicifluum* cv. Hongpigaobachi, triploid *T. vernicifluum* and *T. vernicifluum* cv. Yanggangdamu. The small inversions from *atpF-atpH*, *trnW-trnP*, *trnG-trnR* and *trnQ-psbK* and *rpl16* intron have been occurred in *T. vernicifluum* cv. Dahongpao and *T. vernicifluum* cv. Hongpigaobachi and triploid *T. vernicifluum*. These small inversion regions will provide abundant information for marker development in phylogenetic analyses of related *Toxicodendron* species [48, 52, 53]. However, small inversions of noncoding sequences may influence sequence alignment and character interpretation in phylogeny reconstructions, so caution is necessary when using cp noncoding sequences for phylogenetic analysis [51, 54].

To solve phylogenetic problems at the species level, or to identify species using DNA barcodes, the high evolutionary rates regions were needed to identify [55]. Eight intergenic spacer regions including *trnH-psbA*, *trnK-rps16*, *ycf4-cemA*, *petL-psbJ*, *ndhF-rpl32*, *trnL-ndhD*, *ndhD-ndhE* and one gene regions *ycf1* are highly variable regions in the *Toxicodendron* cp genome. These variable regions may also be useful for assessing phylogenetic relationships and interspecific differences of *Toxicodendron* species [56]. Among these, *trnH-psbA*, *ycf4-cemA* and *ycf1* have been regarded as a candidate DNA barcodes for *Toxicodendron* and other plants [25, 55, 57].

Complete chloroplast genome provides sufficient information sites for resolving phylogenetic relationships of plant, and have been examined to be effective in the ability of differentiation in lower taxonomic levels and provide valuable data for resolving complex evolutionary relationships [58, 59]. Within the Anacardiaceae, Wang et al [25] indicated that *T. vernicifluum* cv. Yanggangdamu was more closely to *R. chinensis* and two *Pistacia* species, due to lack of more *Toxicodendron* cp genomes. In the current study, ten *Toxicodendron* accessions were used to reconstruct the phylogenetic, two distinct clades were recognized: one consisting of *T. vernicifluum* cv. Dahongpao, *T. vernicifluum* cv. Hongpigaobachi, *T. vernicifluum* cv. Yanggangdamu, triploid *T. vernicifluum T. diversilobum* and *T. griffithii* and the other comprised of the remaining accessions. The result is consistent with previous studies, which shown that *T. sylvestre* was more closely related to *T. succedaneum* based on five nuclear and chloroplast DNA regions [60].

Compared to morphological trait classification systems, molecular markers can reveal genetic differences at the DNA reveal genetic differences and are effective for evaluating the genetic diversity of germplasm in breeding programs [61]. SSR markers were testified to be a more advanced tool than all of these markers, which are very suitable for the identification and classification of *Toxicodendron* species [62]. In our study, five accessions can be distinguished clearly by these SSR markers. All the samples of *T. vernicifluum* cv. Dahongpao, *T. vernicifluum* cv. Hongpigaobachi, and triploid *T. vernicifluum* grouped together in one group, and all the samples of *T. vernicifluum* and *T. succedaneum* grouped together in another group, which is consistent with the cp genome and morphological analysis.

## Conclusions

The current study primarily explored the chloroplast genome of triploid *T. vernicifluum* and compared it with related species of *Toxicodendron* genus. The size of genome, structure and organization of gene were shown to be conservative, which is similar to those reported *Toxicodendron* cp genomes. Eight hotspot regions were identified and may be utilized as potential molecular markers for population genetic and phylogenetic studies in *Toxicodendron*. Phylogenetic analysis based on cp genomes and SSR markers both showed that triploid *T. vernicifluum* can be distinguished with *T. vernicifluum* cv. Dahongpao, while *T. vernicifluum* cv. Dahongpao had a close relationship with *T. vernicifluum* cv. Hongpigaobachi. Therefore, complete cp genomes is useful for species identification, taxonomic clarification, and genomic evolutionary analysis. Further research on the relationships within *Toxicodendron* genus and the identification of ploidy should incorporate morphology and genome wide analyses to enhance the results.

## Materials and methods

### Plant materials

In this study, triploid sumac of *T. vernicifluum* cv. Dahongpao (DHP) and triploid *T. vernicifluum* (TZT) were cultivated in the germplasm resource nursery in Yangling Shaanxi Province (108.08E, 34.27N), and Zhaotong Yunnan Province (103.72E, 27.34 N), respectively [11, 13]. Three diploid sumac of *T. vernicifluum* cv. Hongpigaobachi (GBC) were cultivated in the germplasm resource nursery in Yangling Shaanxi Province, *T. succedaneum* (TRB) and *T. vernicifluum* (TZG) were cultivated in the germplasm resource nursery in Southwest Forestry University, in Kunming Yunnan Province (102.76 E, 25.06 N) which were introduced from Changsha Hunan Province and Wenshan Yunnan Province, respectively. These five accessions (DHP, GBC, TZT, TRB and TZG) were used for morphological characterization (Table 1) and SSR molecular markers analysis. GBC, TZT, TRB and TZG were sequenced and combined with the six previously sequenced accessions DHP, *T. succedaneum* (Ts), *T. griffithii* (Tg), *T. sylvestre* (Tsy), *T. diversilobum* (Tdi) and *T. vernicifluum* cv. Yanggangdamu (Tyg) for comparative analysis of chloroplast genome (Table 2).

### Morphological characterization

In order to compare the differences of leaf shapes among the five accessions (DHP, GBC, TZT, TRB and TZG), three trees per accession were selected for data collection on leaves, and five foliar traits (leaf length, leaflet number, leaflet length, leaflet width; and leaf shape index) were measured. All variables were measured using a ruler (0.1 mm resolution) and are recorded in mm and each sample was tested for three biological replicates.

### DNA extraction

Fresh leaves of *T. vernicifluum* cv. Hongpigaobachi (GBC), triploid *T. vernicifluum* (TZT), *T. succedaneum* (TRB) and *T. vernicifluum* (TZG) were collected from different regions and quickly frozen in liquid nitrogen, and stored at ultra-low-temperature refrigerator at -80℃ until use. The voucher specimen deposited in the Herbarium of Southwest Forestry University. The total genomic DNA was extracted with the TGuide plant genomic DNA prep kit (Tiangen Biotech, Beijing, China) and DNA quality was inspected in 0.8% agarose gels, DNA quantification was performed using a NanoDrop spectrophotometer, DNA samples were stored at -80℃ at the Key Laboratory of State Forestry Administration on Biodiversity Conservation in Southwest China, Southwest Forestry University, Kunming, China.

### Plastid genome sequencing, assembly and annotation

Total DNA was used to generate libraries with an average insert size of 350 bp with the Illumina Novaseq 6000 platform. Approximately 8 Gb raw data were produced with 150 bp pair-end read lengths. GetOrganelle software [63] was used to assemble the complete cp genome of the four accessions, with *Pistacia weinmaniifolia* as the reference. Geneious R8 (Biomatters Ltd, Auckland, New Zealand) software was used for initial cp genome annotation. Start and stop condos were checked and adjusted manually when necessary by comparing them to the reference genome *P. weinmaniifolia*. The tRNA genes were further confirmed through online tRNA scane-SE web servers [64]. The gene map of annotated *Toxicodendron* chloroplast genome was drawn by OGdraw online [65]. The annotated sequences have been deposited to the NCBI GenBank database under the accession numbers OP235457, OP271782, OP279729 and OP279730 (Corresponding to *T. succedaneum* (TRB), triploid *T. vernicifluum* (TZT), *T. vernicifluum* cv. Hongpigaobachi (GBC) and *T. vernicifluum* (TZG)).

### Identification of simple sequence repeats (SSRs) and long sequence repeats

Chloroplast SSR loci in ten *Toxicodendron* cp genomes were detected using MISA [66] with the minimal repeat number set to 10, 6, 5, 5, 5 and 5 for mononucleotide (momo-), dinucleotide (di-), trinucleotide (tri-), tetranucleotide (tetra-), pentanucleotide (penta-), and hexanucleotide (hexa-) nucleotide sequences, respectively.

Morever, the online REPuter [67] software was used to estimate and locate forward (F), reverse (R), complemented (C) and palindromic (P) repeats. The following settings for repeat identification were used: (1) Hamming distance equal to 3; (2) minimal repeat size was set to 30 bp; (3) 90% or greater sequence identity.

### Comparative analysis of the chloroplast genomes

To investigate divergence in cp genomes, identity across the whole cp genomes was visualized using the online genome comparison tool mVISTA viewer with Shuffle-LAGAN mode among the ten accessions with *T. vernicifluum* cv. Dahongpao (DHP) [24] as a reference to show inter-and intraspecific variations [68]. The software MAUVE alignment [69] was employed to analyze and compare the plastome structure of triploid *T. vernicifluum* with the other accessions of *Toxicodendron*. Furthermore, events of IR expansion and contraction were compared between these accessions; the junction regions between the IR, SSC, and LSC of ten accessions were compared using the online program IR scope [70].

To identify the mutational hotspot regions for *Toxicodendron*, we calculated nucleotide diversity (Pi) across the whole plastome. The plastome sequences were aligned using MAFFT version 7 software [71] and the nucleotide diversity was detected using DnaSP version 5 software [72] with sliding window strategy. The step size was set to 200 bp, with a 600 bp window length. Single nucleotide polymorphisms (SNPs) were detected using the “find variation” function in Geneious.

### Amplification of SSR marker

The materials used were *T. vernicifluum* cv. Dahongpao (DHP), triploid *T. vernicifluum* (TZT), *T. vernicifluum* cv. Hongpigaobachi (GBC), *T. succedaneum* (TRB) and *T. vernicifluum* (TZG). Three samples per accession were selected for SSR analysis. The fresh leaves from each sample were immediately dried with silica gel for further DNA extraction. Total genomic DNAs were extracted from each sample using the TGuide plant genomic DNA prep kit (Tiangen Biotech, Beijing, China), DNA quality was inspected in 0.8% agarose gels, and DNA quantification was performed using a NanoDrop spectrophotometer. A total of 116 SSR primers [73–77] were used to identify the polymorphic markers among 15 samples, 7 primer pairs amplified well-distributed fragments with good distinctness. Sequence information of the SSRs was listed in Table 5, and the forward of which (F) was labeled 5(6)-carboxyfluorescein (FAM) by the 5’-terminal. PCR reactions were carried out in 25 µL, containing 12.5 µL 2×PCR Mix, 1 µL of each primer, respectively, 1 µL template DNA. The PCR cycling conditions were as follows: pre-denaturation at 94 ℃ for 4 min, followed by 35 cycles of denaturation at 94 ℃ for 30 s, annealing at the corresponding temperature (52 ℃-61 ℃) for 30 s, extension at 72 ℃ for 1 min and the final extension for 10 min at 72 ℃. Amplified SSR alleles were rechecked in 1.5 % binary gels by electrophoresis at 80 volts for 50 min and gel documentation was performed with Gel Logic 200 imaging system. Capillary electrophoresis-based fragment analyses of single pollen amplified SSR alleles were conducted on an ABI3730XL Genetic Analyzer following the manufacture’s instruction to generate GeneScan files. Scoring of alleles was done using ‘GeneMaker’ version 2.2. Genetic distance among the 15 samples was calculated according to the software GenoDive [29] and the cluster analysis was carried out based on the matrix using MEGA 5.0.

**Table 5:** Information of SSR markers

Primers	F(5’-3’)	R(5’-3’)	Repeat motif	T_m_/℃	Sequence length/bp
M156	AAGCTAGCAAATACACATAGG	CTGACAAGTTCCAGACAGGG	(CA)14(CT)9N(AAT/C)16	56	120-152
AG28	TATCGCATCAGGGGTTCCCA	CGGGATGGAGCCGCCAATGA	(GGA)15	61	222-230
M18	AGGCTCCAAATCCATGCCTC	CAAGAGCAAGAACATAGAATATAA	(AAGA)27	52	187-195
B127	GAAGGTGCTAACCCTCTTCTGATA	GCTATGGGGTATTGCTAGATGTTT	(TC)27	54	231-257
B004	TCTGTTACACGTTGCTATTGTACAGG	GATGATGATTCAAACATTCAAACAAAA	(TC)15	57	216-252
B095	TGGGAAGCAACAGTAATCATAGAG	ACTCTTTTCCCTGTTAAAATTTGC	(TC)14	52	163-187
B041	ATTCCCTTCCCCATAAGGATCATTC	TACCTAGTGAGGGAGGAAAAGAGA	(CT)14	56	174-288

### Phylogenetic analysis

Phylogenetic analysis was conducted using the complete chloroplast genome sequences of the ten *Toxicodendron* accessions mentioned above, with two Anacardiaceae species [*Pistacia weinmaniifolia* (MF630953) and *Mangifera indica* (KY635882)] that were used as outgroup. The nucleotide sequences were aligned using MAFFT version 7 software [71]. The phylogenetic analyses were performed using maximum likelihood (ML) and Bayesian inference (BI). ModelFinder [78] was used to select the best-fit model with default setting and the maximum likelihood (ML) analysis was performed using IQ-TREE 1.5.5 [79] with 1000 bootstrap replications. The BI analysis was performed by Mybayes 3.2.6 [80]. The jModelTest 2.0 program [81] was used to determine the best-fitting model for each dataset based on the Akaike information criterion and the optimal model of “GTR+F+R3”. The Markov chain Monte Carlo (MCMC) algorithm was run for 1,000,000 generations, and a burn-in of 25% was used for the analysis.

## Supplementary Information

**Additional file 1: Table S1.** List of genes in the Toxicodendron chloroplast genomes. **Table S2.** SSR repeats in the ten cp genomes. **Table S3.** Ts and Tv in non-coding regions of the nine plastomes, compared with DHP. **Table S4.** Genetic distance among the 15 samples.

## Data Availability

The datasets generated during the current study are available in the NCBI GenBank (Accession number OP235457, OP271782, OP279729, OP279730)
